# Endocan in prediabetes, diabetes, and diabetes-related complications: a systematic review and meta-analysis

**DOI:** 10.1186/s13098-023-01076-z

**Published:** 2023-05-16

**Authors:** Amirmohammad Khalaji, Amir Hossein Behnoush, Behrad Saeedian, Shaghayegh Khanmohammadi, Zahra Shokri Varniab, Soheil Peiman

**Affiliations:** 1 School of Medicine, Tehran University of Medical Sciences, Tehran, Iran; 2 Non-Communicable Diseases Research Center, Endocrinology and Metabolism Population Sciences Institute, Tehran University of Medical Sciences, Tehran, Iran; 3 Pediatric Urology and Regenerative Medicine Research Center, Gene, Cell and Tissue Research Institute, Children’s Medical Center, Tehran University of Medical Sciences, Tehran, Iran; 4 Department of Internal Medicine, AdventHealth Orlando Hospital, Orlando, FL USA

**Keywords:** Diabetes, Meta-analysis, Endocan, ESM-1, Systematic review

## Abstract

**Background:**

Diabetes is one of the chronic conditions with a high burden all around the world. Macrovascular and microvascular involvement are among the common mechanisms by which diabetes can impact patients’ lives. Endocan as an inflammatory endothelial biomarker has been shown to increase in several communicable and non-communicable diseases. Herein, we aim to investigate the role of endocan as a biomarker in diabetes as a systematic review and meta-analysis.

**Methods:**

International databases, including PubMed, Web of Science, Scopus, and Embase were searched for relevant studies assessing blood endocan in diabetic patients. Estimation of the standardized mean difference (SMD) and 95% confidence interval (CI) for comparison of circulating endocan levels between diabetic patients and non-diabetic controls were conducted through random-effect meta-analysis.

**Results:**

Totally, 24 studies were included, assessing 3354 cases with a mean age of 57.4 ± 8.4 years. Meta-analysis indicated that serum endocan levels were significantly higher in diabetic patients in comparison with healthy controls (SMD 1.00, 95% CI 0.81 to 1.19, p-value < 0.01). Moreover, in the analysis of studies with only type-2 diabetes, the same result showing higher endocan was obtained (SMD 1.01, 95% CI 0.78 to 1.24, p-value < 0.01). Higher endocan levels were also reported in chronic diabetes complications such as diabetic retinopathy, diabetic kidney disease, and peripheral neuropathy.

**Conclusion:**

Based on our study’s findings, endocan levels are increased in diabetes, however, further studies are needed for assessing this association. In addition, higher endocan levels were detected in chronic complications of diabetes. This can help researchers and clinicians in recognizing disease endothelial dysfunction and potential complications.

**Supplementary Information:**

The online version contains supplementary material available at 10.1186/s13098-023-01076-z.

## Introduction

Diabetes mellitus is one of the leading health concerns worldwide with a profound impact on public health and socioeconomic development. Despite the decrease in incidence in recent years, diabetes’s prevalence is still increasing in developed countries as well as developing countries [1, 2]. Globally, type 2 diabetes mellitus (T2DM) accounts for almost 90% of the 537 million diabetes cases worldwide [3]. Based on International Diabetes Federation’s reports, 10.5% of adults aged 20–79 had diabetes in 2021, which is expected to grow to 12.2% by 2030 [4].

In addition to being a prevalent chronic disease, diabetes poses microvascular and macrovascular complications [5]. By early diagnosis and treatment, healthcare systems can reduce microvascular and macrovascular complications of diabetes which can lead to improvement in the disease’s outcome [3, 6, 7]. Moreover, in light of the high prevalence of T2DM, non-specific or only partial symptoms in the early stages, early diagnosis is particularly essential, leading to intensive studies on identifying a novel biomarker for T2DM such as endocan [5].

While T1DM is a result of autoimmune destruction and T2DM is mainly driven by β-cell dysfunction and insulin resistance [8, 9], an association is observed between diabetes mellitus and endothelial dysfunction [10]. Recent researches suggest that the endothelial and insulin signaling pathways interact, resulting in impaired vascular response and nitric oxide-dependent vasodilation, reduced cellular uptake of glucose, enhanced oxidative stress, and inflammation. As a result of all these pathophysiologic mechanisms, atherosclerosis could develop [11]. In addition to being a key factor in the development of atherosclerosis [12], endothelial dysfunction plays a critical role in its progression. In addition, it is an early indicator of diabetic vascular disease that can independently predict the cardiovascular risk [10, 13].

Previously called endothelial cell-specific molecule-1 (ESM1), endocan may be indicative of endothelial dysfunction [14]. It is a soluble dermatan sulfate proteoglycan secreted and expressed predominantly by vascular endothelial cells but can also be found in serum and plasma [15, 16]. Endocan regulates endothelium activation, permeability, and proliferation [17, 18]. Since endocan affects inflammatory and vasculoprotective signals, it might be effective in atherosclerosis and is an endothelial dysfunction marker [17]. Endocan levels have been reported to be higher in patients with endothelial damage and neovascularization, whereas normal levels are found in patients with functioning endothelial tissue [19, 20].

In this article, we reviewed the role of endocan as an endothelial marker in prediabetes, diabetes, and diabetes-related complications (retinopathy, nephropathy, and neuropathy) in addition to its diagnostic utility in special populations of diabetes (e.g., cardiovascular diseases and obstructive sleep apnea). Moreover, we compared the serum levels of endocan in diabetics with non-diabetic subjects and T2DM with non-diabetics using meta-analysis.

## Methods

### Search strategy

PRISMA (Preferred Reporting Items for Systematic Reviews and Meta-analysis) statement was used for the conduction of the current systematic review and meta-analysis [21]. The following databases were searched from inception through February 13, 2023, with no restrictions or any filters: PubMed, Embase, Scopus, and the Web of Science. The search terms used in our study were: “diabetes” OR “diabetic” OR “pre-diabetes” OR “prediabetic” AND “Endocan” OR “ESM-1” OR “endothelial cell-specific molecule 1”. The search strategy and all the used keywords are explained in detail in Supplementary Table 1. Two independent reviewers (AK and AHB) systematically reviewed all studies with title and abstract for inclusion and the full text for the primary review. In cases of disagreements, the conclusion was finalized by a discussion with the third reviewer (BS).

### Study selection

The applied inclusion criteria were (1) clinical studies that measured the blood level of endocan in patients with diabetes and compared them with the control group; (2) studies that evaluated the blood level of endocan in pre-diabetic patients and compared them with the control group. Exclusion criteria were as follows: (1) not reported endocan levels or exact endocan levels; (2) reported endocan levels in mediums other than blood (such as vitreous or gingival crevicular fluid); (3) conference abstracts, letters, or review articles.

We defined the PICO (population, intervention, control, and outcome) for selecting studies as:

(P): patients with diabetes, prediabetes, or diabetes-related complications.

(I): measuring circulating endocan levels as a biomarker in patients and controls.

(C): healthy individuals or diabetic patients without chronic comorbidities.

(O): could the peripheral endocan level significantly differentiate patients with prediabetes and diabetes from healthy individuals or the cases with diabetes-related complications from the ones without chronic complications.

### Data extraction

Data extraction of the included studies was carried out by one of the reviewers (BS) and cross-checked by a second reviewer (AK). We extracted the following data: (1) first author name, publication year, publication country, and design of the study; (2) study population, the definition of diabetic and control groups; (3) type of diabetes in diabetic groups, existing diabetes complications, and comorbidities; (4) the number of participants in each group, age mean ± standard deviation (SD), sex proportions, and HbA1c mean and SD in total population; (5) main findings; and (6) plasma and/or serum endocan levels.

### Quality assessment

The methodological quality of the studies was assessed by two reviewers (AK and AHB), applying the “Newcastle–Ottawa Quality Assessment Scale” (NOS) checklist [22] for cohort and case-control studies and Downs and Black guidelines for cross-sectional studies [23]. According to NOS, selection, comparability, and outcome were assessed as potential sources of bias. Each of them was categorized as “very good,” “good,” “satisfactory,” or “unsatisfactory” based on the scores of 9–10, 7–8, 5–6, and < 5, respectively. Regarding the Downs and Black system, we used the checklist customized for our included studies which are observational in nature. Hence, we only assessed items 1, 2, 3, 6, 7, 10, 11, 12, 18, 20, 21, and 22. Each item can be scored as 1 for a “Yes” answer and 0 for a “No”/”unable to determine” answer. As suggested by Ratcliffe and collaborators [24], the overall qualities of the studies were graded as “high quality” by achieving a score of > 66.8% (> 8), “medium quality” with a score of 33.4–66.7% [4–8], and “low quality” by a total score of < 33.3% (< 4). Quality assessment was performed by two independent reviewers (AK and AHB) and the third reviewer (BS) solved any disagreements between the two reviewers. Kappa Cohen’s [25] was also calculated for the assessment of agreement between the two independent reviewers.

### Statistical analysis

Random-effect model was used for the conduction of meta-analysis. We calculated the estimation of standardized mean difference (SMD) in addition to 95% confidence interval (CI) for comparison between endocan levels in diabetic patients and controls. All the analyses were done using STATA (version 17.0, Stata Corp), and a p-value < 0.05 was considered statistically significant. We also assessed the quality of evidence and strength of recommendations based on the GRADE approach with incorporates five domains: risk of bias, inconsistency, indirectness, imprecision, and publication bias [26].

In cases of endocan levels reported in median and interquartile range or median and range, we used Luo et al. [27] and Wan et al. [28] methods to convert those data into median and SD. Using Cochrane’s Q and Higgin’s I^2^ test, the heterogeneity of studies was calculated. The considered heterogeneity thresholds were: ≤ 25% for low, 26–75% for moderate, and > 75% for high [29]. We conducted meta-regression based on mean age, publication year, sample size, male percentage, and HbA1c Supplementary Figs. 2–6, and subgroup analysis in regard to diabetes type and comorbidities, both in diabetic patient groups. Finally, statistical tests of Egger’s [30] and Begg’s [31] in addition to the funnel plot visual assessment were utilized to recognize publication bias.

## Results

### Literature search and included studies characteristics

**Fig. 1:**
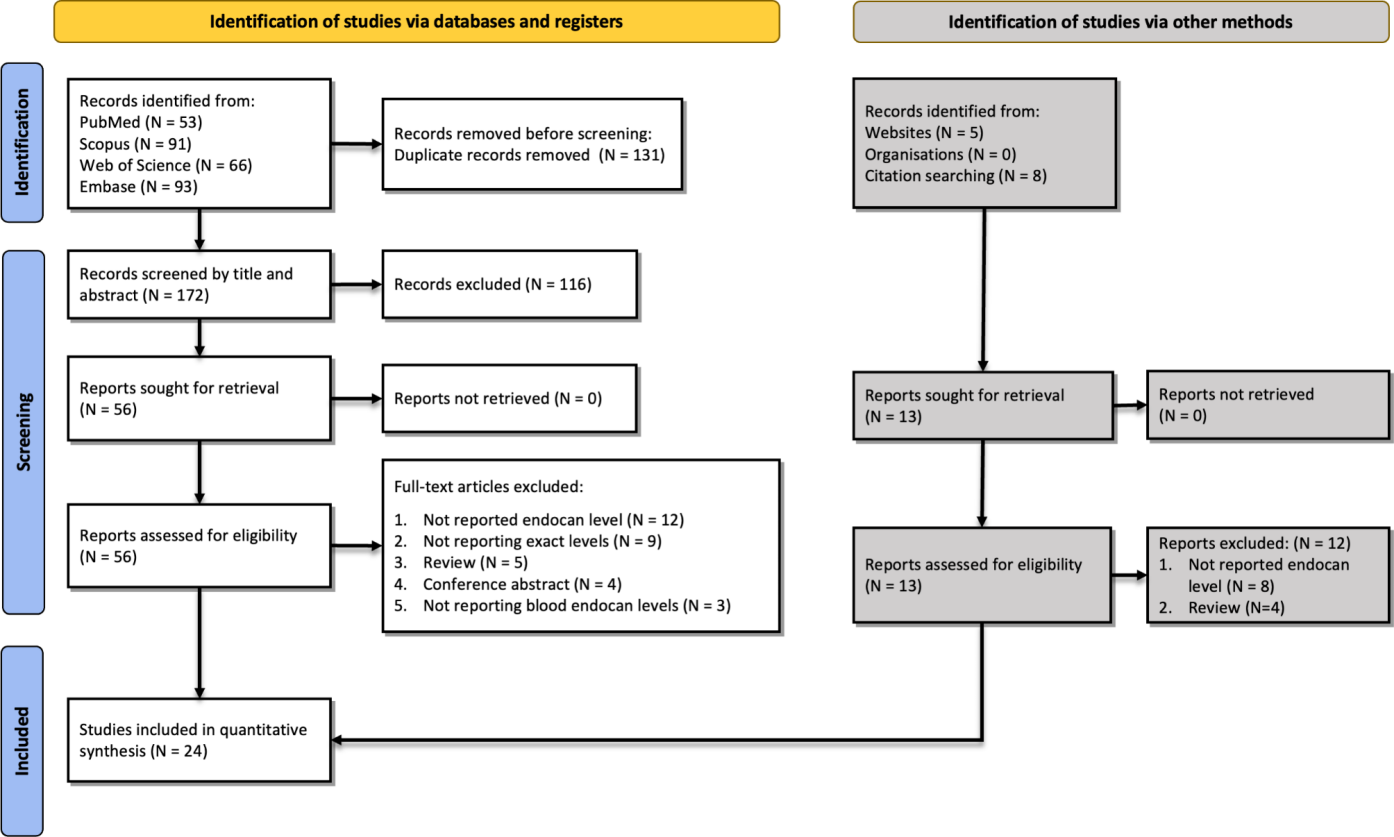
PRISMA flowchart summarizing the selection process of eligible studies based on inclusion/exclusion criteria

The initial search yielded 303 results: 53 from PubMed, 66 from Web of Science, 91 from Scopus, and 93 from Embase. After the removal of the duplicates (n = 131), 172 studies remained. Title/abstract screening resulted in 56 remaining studies and full-text screening led to the exclusion of 33 studies. Manual searching also resulted in 5 studies from websites and 8 from citation searching, among which one was finally included. The most frequent reason for exclusion both in database searches screening and manual search was not reporting endocan levels. Details and flowchart of searching and exclusion reasons are shown in Fig. 1.

Finally, 24 studies were included and their characteristics are described in Table 1 [32–55]. A total of 3354 patients with a mean age of 57.35 ± 8.35 years and 52.56% were male. Other than five studies in which “plasma” endocan was reported, most studies measured “serum” endocan levels [39, 40, 46, 50, 52]. One study included T1DM patients [32]; however, the majority of studies had only T2DM as their included population [33, 35, 36, 38, 39, 42, 44, 45, 47, 48, 51–53, 55]. All cohort and case-control studies were of high quality based on the NOS scoring system (Supplementary Table 2). Cross-sectional studies also had high qualities based on our customized Downs and Black criteria, except for Bilir et al. [36] that had a score of 7 in the overall quality assessment (Supplementary Table 3). The agreement percentage was 87.5% and Cohen’s k was 0.75 for independent quality assessments by two authors.

**Table 1:** Characteristics of studies evaluating endocan levels in diabetic patients

Author	Year	Design	Location	Specimen	Population	DM type	Special population	N total	Age	% Male	Main Findings
Anik et al.	2020	Cross-sectional	Turkey	Serum	Children with T1DM and age-, gender-, and pubertal stage distribution-matched healthy children	T1DM	No	128	12.0 ± 3.7	47.6	Serum levels of endocan were higher in T1DM children than in healthy ones (P < 0.01) and there was a significant positive correlation between endocan and serum HbA1c (r = 0.296, P = 0.01).
Arman et al.	2015	Prospective cohort	Turkey	Serum	Patients diagnosed with T2DM and healthy controls	T2DM	No	83	55.4 ± 10.2	42.2	Serum endocan was significantly higher in patients with T2DM than in healthy controls (1.56 ± 0.99 ng/ml vs. 0.72 ± 0.1 ng/ml, P < 0.001). After 3 months of treatment, there was a significant reduction in HbA1c (10.7 ± 2.28% vs. 7.57 ± 1.17%, P < 0.0001) and endocan levels (1.55 ± 0.99 ng/ml vs. 1.07 ± 0.71 ng/ml, P < 0.0001). However, endocan levels were still higher than healthy controls (1.07 ± 0.71 ng/ml vs. 0.72 ± 0.1 ng/ml, P < 0.001).
Arman et al.	2022	Cross-sectional	Turkey	Serum	Patients with diabetes and healthy participants	Pre-DM	No	84	48.8 ± 9.1	31.0	Endocan was lower in prediabetes patients than in healthy patients (P = 0.042) and there was a negative correlation between fasting insulin and levels of endocan (r=-0.320, P = 0.001).
Balamir et al.	2017	Cross-sectional	Turkey	Serum	Patients in the 18 to 65 age groups under constant follow-up in a clinic with T2DM diagnosis and healthy controls	T2DM	No	176	54.3 ± 9.9	35.8	Median serum endocan was higher in T2DM patients and endothelial damage than those in T2DM patients without endothelial damage (475.15 pg/ml vs. 216.37 pg/ml, P < 0.001, respectively).
Bilir et al.	2016	Cross-sectional	Turkey	Serum	Diabetic patients with or without DPN and healthy controls	T2DM	No	152	57.7 ± 8.4	46.7	Diabetic patients (with or without DPN) had higher endocan than healthy controls (P < 0.001). Endocan levels were found to be significantly higher in diabetic patients with DPN than those without DPN (P = 0.001). When grouping patients according to treatment, those who received insulin had higher endocan levels than those who received oral antidiabetic medications (P = 0.004).
Bingol et al.	2016	Prospective cohort	Turkey	Serum	Subjects with suspicion of OSA	DM	OSA	63	49.6 ± 9.5	52.4	Serum levels of endocan seem to be higher in OSA patients with diabetes than those without it, but the difference is not significant (1.48 ± 0.86 vs. 1.19 ± 0.3 ng/ml, P = 0.489).
Bozkurt et al.	2020	Cross-sectional	Turkey	Serum	Patients with DM who were admitted to the Department of Ophthalmology and control patients without any systemic disease	T2DM	No	100	61.9 ± 8.3	50.0	Serum endocan levels increase with the stage of diabetic retinopathy and it was found to be an independent predictor showing the stage.
Celik et al.	2022	Cross-sectional	Turkey	Plasma	Patients who underwent Phacoemulsification surgery in an ophthalmology clinic and controls without any medical problems	T2DM	No	120	71.2 ± 5.2	54.2	Blood endocan was higher in patients with DM and cataracts, DRP and cataracts, and patients with cataracts than in healthy individuals.
Chang et al.	2021	Prospective cohort	Taiwan	Plasma	Patients with T2DM and regular visitors of the outpatient department of the Division of Endocrinology and Metabolism	DM	No	312	62.3 ± 11.9	68.0	It was found that plasma endocan levels were not related to the occurrence of renal events in patients with diabetes.
Chen et al.	2022	Cross-sectional	China	Serum	Patients with DKD	DM	DKD	183	58.0 ± 8.5	68.3	Serum endocan levels were negatively correlated with HbA1c (r = -0.21, P < 0.01) and estimated GFR (r = -0.206, P < 0.01) and positively correlated with 24 h urine protein (r = 0.219, P < 0.01).
Cikrikcioglu et al.	2016	Cross-sectional	Turkey	Serum	Patients with T2DM attending to an internal medicine outpatient clinic	T2DM	No	137	56.4 ± 8.4	30.6	Serum endocan levels were negatively correlated with the urine albumin-creatinine ratio (r = 0.282, P = 0.001). Patients with or without retinopathy and with or without neuropathy were comparable in terms of serum endocan levels. There was no correlation between serum endocan and diabetes duration, BMI, eGFR, HbA1c, and fasting glucose.
Dallio et al.	2017	Cross-sectional	Italy	Serum	Consecutive NAFLD patients with or without T2DM and healthy subjects	DM	NAFLD	81	56.6 ± 11.2	53.0	In patients with NAFLD, diabetic patients had significantly higher endocan compared to non-diabetic ones (1.56 ± 0.81 ng/ml vs. 0.72 ± 0.58 ng/ml, P = 0.001).
Ekiz-Bilir et al.	2019	Cross-sectional	Turkey	Serum	Diabetic patients admitted to hospital and healthy controls who were admitted to internal medicine out-patient clinics for a routine medical assessment	T2DM	No	131	56.1 ± 7.8	53.4	Serum endocan levels were higher in diabetic patients with nephropathy than those with normoalbuminuria (P = 0.011) and both groups had higher serum endocan levels compared with healthy individuals (P < 0.001 and P = 0.001, respectively).
Elkamshoushi et al.	2018	Cross-sectional	Egypt	Serum	Patients with ED who were recruited from Andrology Outpatient Clinic and healthy controls	T2DM	ED	45	41.8 ± 4.8	100	Serum endocan levels in ED patients with DM were significantly higher than patients without DM (P = 0.013) and both groups showed significantly higher levels than healthy individuals (P < 0.001 and P = 0.001, respectively).
Kim et al.	2020	Prospective cohort	Korea	Plasma	Patients with ESRD on hemodialysis	DM	ESRD	354	62.1 ± 12.7	NR	In patients with ESRD, patients with lower endocan levels had a higher rate of DM (64.6%) compared to the higher endocan group (47.2%) (P = 0.001).
Klisic et al.	2020	Cross-sectional	Montenegro	Serum	Patients with prediabetes and T2DM patients, compared with healthy controls	T2DM	No	278	61.5 ± 3.2	39.2	T2DM patients had significantly higher endocan compared to healthy individuals and there was no significant difference between T2DM and prediabetic patients. It was found that a rise in endocan levels by one level increases the probability of a higher HbA1c by three times (OR 3.69, 95% CI 1.84 to 7.01, P < 0.001).
Klisic et al. (2)	2020	Case-control	Montenegro	Serum	Patients with T2DM and diabetes-free participants	T2DM	No	106	61.8 ± 10.0	44.3	Serum endocan levels were higher in diabetic patients in comparison to healthy individuals (P = 0.005).
Kose et al.	2015	Cross-sectional	Turkey	Serum	Patients who were diagnosed as having ACS, control group (age- and sex-matched)	DM	ACS	83	56.0 ± 10.6	78.3	Endocan levels were higher in diabetic patients with ACS than non-diabetic patients with ACS (1.02 ± 0.33 ng/ml vs. 0.81 ± 0.21 ng/ml, P = 0.016). Both groups showed a significant difference compared with controls (0.86 ± 0.25 ng/ml vs. 0.75 ± 0.13 ng/ml, P = 0.014).
Kosir et al.	2019	Prospective cohort	Slovenia	Plasma	Consecutive chronic HF patients	DM	HF	120	71.0 ± 11.0	64.0	Plasma endocan levels had no significant difference between diabetic and non-diabetic heart failure patients (P > 0.05).
Lv et al.	2017	Cross-sectional	China	Serum	Patients with T2DM and gender-matched and age-matched healthy subjects	T2DM	No	97	50.3 ± 9.8	58.6	Endocan was significantly higher in diabetic patients with subclinical atherosclerosis than diabetic patients than healthy controls (P < 0.05 for all comparisons).
Moin et al.	2022	Case-control	Bahrain	Plasma	Subjects with T2DM and nondiabetic control Caucasian subjects, all aged 40–70 years	T2DM	No	46	62.0 ± 9.0	50.0	Plasma endocan levels were lower in T2DM patients than healthy controls (P < 0.05).
Qiu et al.	2016	Cross-sectional	China	Serum	Patients with T2DM and normotensive control participants	T2DM	No	105	63.1 ± 10.0	55.2	Endocan peripheral levels were significantly higher in T2DM patients with STEMI than T2DM without STEMI and it was significantly higher in both groups compared with controls.
Singh et al.	2022	Prospective cohort	India	Serum	Diabetic patients with dengue fever and non-diabetic patients with dengue fever	DM	Dengue fever	40	56.5 ± 3.0	NR	Endocan circulatory levels were significantly higher in dengue fever and diabetes than those with dengue fever without diabetes (P < 0.0001).
Zuwala-Jagiello et al.	2019	Retrospective cohort	Poland	Serum	Patients treated for liver cirrhosis and control serum samples were collected from age- and gender-matched healthy subjects in whom liver diseases were ruled out	T2DM	Cirrhosis	330	NR	54.2	Serum concentrations of endocan were significantly higher in cirrhosis patients compared with healthy individuals (P < 0.001) and cirrhotic patients with diabetes had higher endocan blood levels than cirrhotic non-diabetic patients (P < 0.01).

Data are presented as mean ± standard deviation, median [interquartile range], median [range], or percentage. DM: diabetes mellitus; T1DM: type 1 diabetes mellitus; T2DM: type 2 diabetes mellitus; DPS: diabetes peripheral neuropathy; OSA: obstructive sleep apnea; NAFLD: non-alcoholic fatty liver disease; DKD: diabetic kidney disease; ED: erectile dysfunction; ESRD: end-stage renal disease; ACS: acute coronary syndrome; NR: not reported; DRP: diabetic retinopathy; GFR: glomerular filtration rate; BMI: body mass index; OR: odds ratio; CI: confidence interval; HF: heart failure; STEMI: ST-elevated myocardial infarction

### Meta-analysis

#### Meta-analysis of endocan levels in serum in diabetic patients vs. healthy controls

**Fig. 2:**
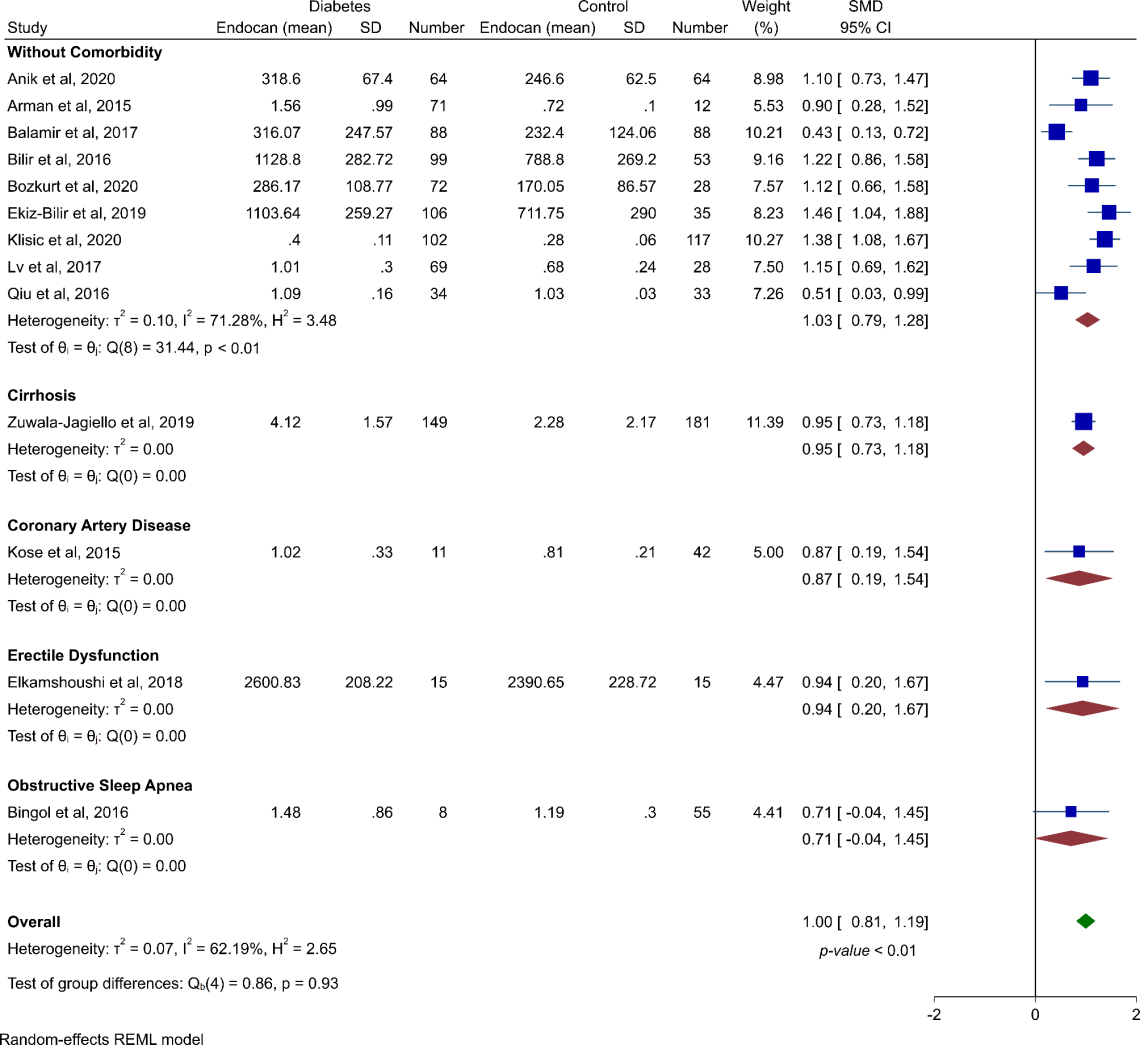
Forest plot showing meta-analysis and subgroup analysis of serum endocan levels in diabetic patients vs. healthy controls

Thirteen studies reported exact endocan levels in diabetic patients and non-diabetic ones and were included in the meta-analysis. Meta-analysis of endocan levels in diabetic patients vs. non-diabetic cases showed that there is a significantly increased level of endocan in diabetes (SMD 1.00, 95% CI 0.81 to 1.19, p-value < 0.01). The heterogeneity was moderate in this meta-analysis (*I*^*2*^: 62.19%). The forest plot showing this meta-analysis is illustrated in Fig. 2.

Four studies investigated patients with other diseases than diabetes, including obstructive sleep apnea [37], erectile dysfunction [45], coronary artery disease [49], and cirrhosis [55]. A subgroup analysis was performed for other studies without comorbidities and as shown in Fig. 2, there were increased serum endocan levels in these patients in comparison with healthy controls (SMD 1.03, 95% CI 0.79 to 1.28, p-value < 0.01, *I*^*2*^: 62.19%).

#### Publication bias assessment, meta-regression, and quality of evidence

Visual assessment of the funnel plot showed no significant source of publication bias (Supplementary Fig. 1). Similarly, Begg’s and Egger’s tests also did not indicate any sign of publication bias (p-value = 0.246 and p-value = 0.604, respectively). Meta-regression showed that none of the mean age, publication year, male percentage, sample size, and HbA1C levels had an association with the SMD of meta-analysis. Moreover, the publication year accounted for 23.01% of heterogeneity, and levels of HbA1C had R^2^ of 4.91% (Table 2). The bubble plots for these analyzes are illustrated in Supplementary Figs. 2–6. GRADE approach also revealed a moderate quality of analyses, due to high inconsistency which stems from the high heterogeneity observed (Table 3).

**Table 2:** Meta-regression of endocan levels in patients with diabetes mellitus vs. controls

Moderator	No. of Comparisons		Meta-regression				R^2^ Analog (proportion of variance explained)
	Diabetes	Control	Slope	95% confidence interval		p-value	
**Mean Age (years)**	888	751	-0.0007	-0.0153	0.0138	0.923	0%
**Mean HbA1c (percentage)**	671	425	-0.152	-0.4493	0.1453	0.316	4.91%
**Publication Year**	888	751	0.0837	-0.0105	0.1779	0.082	23.01%
**Male sex (percentage)**	888	751	0.0002	-0.0134	0.0138	0.973	0%
**Sample Size**	888	751	0.0006	-0.0178	0.003	0.615	0%

**Table 3:** Summary of the GRADE quality of evidence assessment

Quality Assessment						Number of Patients		SMD (95% CI)	Quality
# Studies	Risk of Bias	Inconsistency	Indirectness	Imprecision	Publication Bias	DM	Control		
DM vs. Healthy Controls									
13	Not serious	Serious	Not serious	Not serious	Not serious	888	751	1.00 [0.81, 1.19]	⊕⊕⊕Ο Moderate^†^
**DM without other comorbidities vs. Healthy Controls**									
9	Not serious	Serious	Not serious	Not serious	Not serious	705	458	1.03 [0.79, 1.28]	⊕⊕⊕Ο Moderate^†^
**T2DM vs. Healthy Controls**									
10	Not serious	Serious	Not serious	Not serious	Not serious	641	394	1.01 [0.20, 1.67]	⊕⊕⊕Ο Moderate^†^
**T2DM without other comorbidities vs. Healthy Controls**									
8	Not serious	Serious	Not serious	Not serious	Not serious	805	590	1.02 [0.74, 1.31]	⊕⊕⊕Ο Moderate^†^

Abbreviations: CI: confidence interval, SMD: Standardized Mean Difference

†Moderate due to serious inconsistencies and high heterogeneity in meta-analysis

#### Meta-analysis of serum endocan levels in type 2 diabetes vs. healthy control

**Fig. 3:**
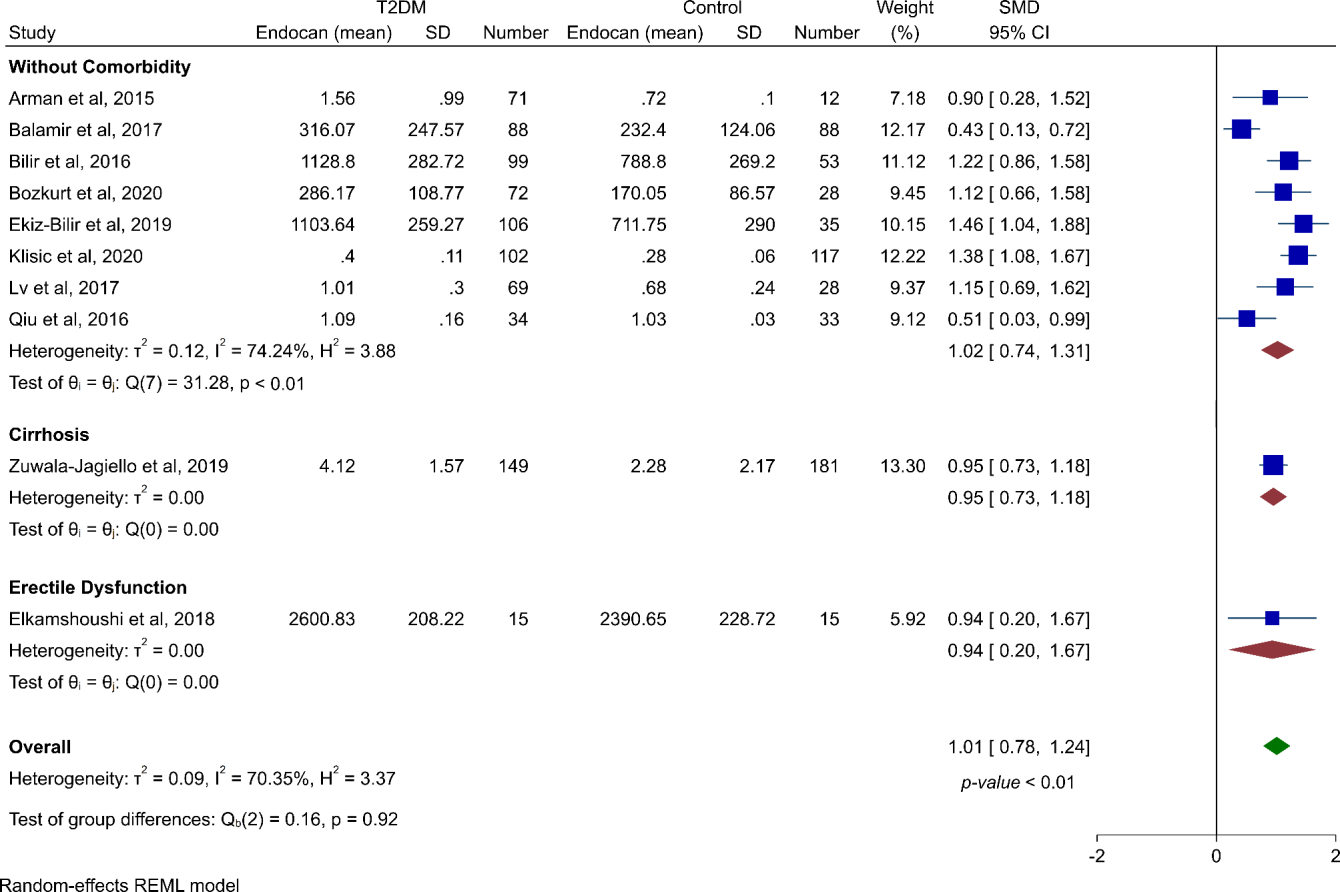
Forest plot showing meta-analysis and subgroup analysis of serum endocan levels in type 2 diabetic patients vs. healthy controls

Meta-analysis showed that endocan is statistically higher in type 2 diabetic patients (SMD 1.01, 95% CI 0.78 to 1.24, p-value < 0.01) (Fig. 3) in spite of the fact that this was associated with moderate heterogeneity (*I*^*2*^: 70.35%). Analysis in a subgroup of studies including patients without comorbidity resulted in the same result (SMD 1.02, 95% CI 0.74 to 1.31, p-value < 0.01). Table 3 shows that the evidence assessment of these two analyses had moderate quality.

### Endocan in pre-diabetic patients vs. controls

Two studies investigated circulatory endocan levels in pre-diabetic patients [34, 47]. Arman et al. [34] compared endocan levels between 42 pre-diabetic and 42 healthy controls and found significantly decreased levels of endocan in patients with pre-diabetes (120 [65–185] ng/l vs. 138 [84–300] ng/l, p-value = 0.042). However, Klisic et al. [47] found comparable endocan levels between pre-diabetic patients and healthy controls (pre-diabetes: 0.308 [0.248–0.383] ng/ml vs. control: 0.282 [0.246–0.323] ng/ml; p-value > 0.05). Patients with T2DM had higher levels of endocan compared to both pre-diabetic patients and healthy controls (P < 0.01).

### Endocan levels in complications of diabetes

#### Kidney diseases

Chen et al. [41] measured peripheral endocan in patients with diabetic kidney disease (DKD) and divided them into three groups based on proteinuria and estimated glomerularfiltration rate (eGFR): early DKD, established DKD, and advanced DKD. The early DKD group had significantly lower endocan levels (688.76 ± 274.71 pg/ml) compared to both established (691.62 ± 293.39 pg/ml) and advanced DKD groups (739.78 ± 325.70 pg/ml) (p-value < 0.05). In addition, advanced DKD was associated with statistically higher levels of endocan in comparison with established DKD (p-value < 0.05). A study conducted by Chang et al. [40] compared renal events between tertiles of endocan levels in patients with T2DM. They found no association between the occurrence of renal events and endocan levels in this prospective cohort.

The relation between albuminuria and levels of endocan in diabetic patients was investigated in two studies [42, 44]. Cikrikcioglu et al. [42] divided patients with T2DM into normo-albuminuria, microalbuminuria, and macroalbuminuria groups. They found significantly lower endocan in patients with macroalbuminuria in comparison with normo-albuminuric ones (379.96 ± 189.95 ng/l vs. 495.45 ± 344.82 ng/l; p-value = 0.039). Other comparisons between these groups resulted in insignificant differences (p-value > 0.05). In another study, Ekiz-Bilir et al. [44] found significantly higher endocan levels in patients with normo-albuminuria (1011.4 [429.9–1681.8] ng/l) and nephropathy (1175.3 [564.5–1637.5] ng/l) compared to healthy controls (680.77 [213.3–1433.1] ng/l) (p-value = 0.001 and p-value < 0.001, respectively). Moreover, patients with nephropathy had higher levels of endocan than normo-albuminuric patients (p-value = 0.011).

#### Retinopathy

Bozkurt et al. [38] compared endocan levels between T2DM patients without retinopathy (G2, n = 21), non-proliferative T2DM retinopathy patients (G3, n = 24), proliferative T2DM retinopathy (G4, n = 27), and healthy controls (G1, n = 28). Endocan levels were meaningfully higher in diabetic patients in comparison with non-diabetic ones and the levels were also higher in proliferative retinopathy than in non-proliferative retinopathy (G1: 170.05 ± 85.67 ng/l, G2: 333.91 ± 13.41, G3: 340.42 ± 105, G4: 472.83 ± 147; p-value < 0.05 in One Way Anova). In a study conducted by Celik et al. [39], they found significantly higher levels of endocan in diabetic patients with retinopathy and cataract compared to diabetic patients with cataracts and without retinopathy (7.69 ± 0.39 ng/ml vs. 6.58 ± 0.50 ng/ml, p-value < 0.01).

#### Neuropathy

In a single-blind controlled trial conducted by Bilir et al. [36], diabetic patients with peripheral neuropathy had significantly higher, compared to diabetic patients without neuropathy (1227.1 [575.9–1862.3] vs. 1043.0 [429.9–1678], p-value < 0.001) and healthy controls (1227.1 [575.9–1862.3] vs. 781.8 [213.3–1433.1], p-value < 0.001). Moreover, diabetic patients without neuropathy had significantly higher levels of endocan compared to healthy controls (1043.0 [429.9–1678] vs. 781.8 [213.3–1433.1], p-value < 0.001).

### Endocan in special populations of diabetic patients

#### Cardiovascular diseases

Four studies evaluated circulatory endocan in diabetic patients with or without cardiovascular comorbidity [35, 49, 51, 53]. Kose et al. [49] found significantly higher levels of endocan in patients presenting with acute coronary syndrome (ACS) with diabetes compared to ACS patients without diabetes (1.02 ± 0.33 ng/ml vs. 0.81 ± 0.21 ng/ml, p-value = 0.016). A study by Lv et al. [51] compared endocan levels between diabetic patients (with or without subclinical atherosclerosis) and healthy controls. They found higher endocan concentrations in diabetic patients with subclinical atherosclerosis (1.20 ± 0.33 ng/ml) compared to diabetic patients without subclinical atherosclerosis (0.89 ± 0.28 ng/ml, p-value < 0.05) and healthy controls (0.68 ± 0.24 ng/ml). In another study by Qiu et al. [53], diabetic patients presented with ST-elevation myocardial infarction (STEMI) had higher endocan levels compared to diabetic patients without STEMI (1.25 ± 0.50 ng/ml vs. 1.09 ± 0.16, p-value < 0.05) and healthy controls without cardiovascular complications (1.03 ± 0.03 ng/ml, p-value < 0.05). Finally, Balamir et al. [35], investigated endocan levels in diabetic patients with or without endothelial dysfunction (defined by carotid intima-media thickness) and healthy controls. They found significantly higher endocan levels in diabetic patients with endothelial dysfunction compared to diabetic patients without endothelial dysfunction (475.1 [123.7–1274.6] pg/ml vs. 216.4 [60–731.6] pg/ml, p-value < 0.001).

#### Obstructive sleep apnea

Bingol et al. [37] evaluated endocan in patients with obstructive sleep apnea (OSA) with and without diabetes. They found comparable levels of endocan between OSA patients with and without diabetes (1.48 ± 0.86 ng/ml vs. 1.19 ± 0.3 ng/ml, p-value = 0.489).

#### Liver diseases

Dallio et al. [43] evaluated endocan levels in patients with non-alcoholic fatty liver disease (NAFLD) patients, diabetic patients, and healthy controls. Among patients with NAFLD, diabetic patients had significantly higher endocan levels compared to non-diabetic ones (1.56 ± 0.81 ng/ml vs. 0.72 ± 0.58 ng/ml, p-value = 0.001). Moreover, in another study by Zuwala-Jagiello et al. [55], patients with cirrhosis and diabetes had significantly higher endocan levels compared to non-diabetic patients with cirrhosis (4.08 [3.1–5.2] ng/ml vs. 2.6 [0.7–3.6], p-value < 0.01).

#### Erectile dysfunction

Elkamshoushi et al. [45] evaluated endocan levels in patients with erectile dysfunction (with or without T2DM) and healthy controls. Endocan levels were significantly higher in diabetic patients with erectile dysfunction compared to non-diabetic patients with erectile dysfunction (2600.83 ± 208.22 ng/ml vs. 2390.65 ± 228.72 ng/ml, p-value = 0.013).

## Discussion

In the current study, we compared the level of endocan in diabetic patients and non-diabetic cases. Our meta-analysis showed that the level of serum endocan is significantly increased in patients with diabetes compared to non-diabetic controls. Patients with T2DM also have increased levels of endocan compared with healthy controls. Besides, endocan levels in neuropathy, retinopathy, or cardiovascular diseases are higher than in diabetic patients without these complications. Overall, it seems that endocan might be a possible suitable candidate for the assessment of endothelial dysfunction in diabetic patients.

At the molecular level, endocan is a soluble proteoglycan primarily released by endothelial cells. Its expression is up-regulated by inflammatory markers, including tumor necrosis factor-α (TNF-α) and interleukin (IL)-1β, which in turn leads to the higher expression of vascular cell adhesion molecule 1 (VCAM-1) and intercellular adhesion molecule 1 (ICAM-1); leukocyte migration and inflammatory response are the result of the higher expression of these cell adhesion molecules. In line, endocan levels are also elevated in other conditions, such as malignancies, inflammatory diseases, hypertension, atherosclerosis, carotid artery disease, peripheral artery disease, and sepsis [56]. Therefore, it is not surprising that it is increased in diabetes due to its inflammatory role same as other diseases.

Another rationale by which endocan might be increased is endothelial dysfunction observed in diabetes as well as other diseases. Increased plasma levels of endocan are thought to be a possible immuno-inflammatory marker that may represent endothelial activation and dysfunction and may be linked to diseases causing endothelial damage like diabetes [14, 57]. Based on experimental and clinical studies, there is a link between insulin resistance and endothelial dysfunction, for which newer anti-diabetic agents are modified to target it [58]. Moreover, since atherosclerotic events are one of the main pathways diabetes can affect health, considering the highlighted role of the endothelium in its progression [59], endocan could be suggested as a prognostic biomarker. Interestingly, endothelial dysfunction has been also reported in prediabetic conditions like impaired fasting glucose (IFG) and impaired glucose tolerance (IGT) [60].

Based on our study’s findings, endocan is raised in diabetes-related complications such as diabetic retinopathy, neuropathy, and kidney disease. Vascular and endothelial damage in diabetic patients is correlated with many complications, such as retinopathy [61], neuropathy [62], and cardiovascular diseases [63]. As our results showed that the increased endocan levels are associated with the complications mentioned above, endocan could be used as a predictive factor for complications in diabetic patients. The association of retinopathy, neuropathy, and cardiovascular diseases with endocan levels could be explained by its role in endothelial activation, permeability, and proliferation, as well as its association with endothelial dysfunction [56]. Moreover, it has been recommended that the mechanism by which diabetes can affect macro- and microvasculature, and hence these complications, is through the release of pro-inflammatory cytokines and free radicals [64] as mentioned earlier. In the study by Abu El-Asrar et al., vitreous fluids of diabetic patients with active proliferative retinopathy were compared with those of controls. It was shown that vitreous endocan levels were higher in retinopathic patients [65]. In addition to retinopathy, diabetic macular edema, one of the most important causes of visual impairment, is also reported to be associated with an increase in blood endocan [38].

Several studies have shown a diagnostic role for endocan in kidney diseases, such as acute kidney injury (AKI), chronic kidney disease (CKD), and renal replacement therapy (hemodialysis or kidney transplantation); however, the results are conflicting, and the exact mechanism of endocan in kidney function has not fully determined [66]. Results regarding the association of endocan levels with albuminuria and DKD in diabetic patients were controversial, and more studies are required to determine the association of endocan levels with kidney diseases and their progression in patients with diabetes.

The fact that endocan was mostly increased in diabetic patients with other diseases compared with controls shows that endocan is more severely related to diabetes rather than these diseases. So, endocan is still a useful biomarker of diabetes in these patients. For sure, other studies with larger sample sizes are needed to confirm these findings.

Considering all these findings, a point of caution is that increased endocan levels could not be the only determining marker in the diagnosis of diabetes and predicting its future complications. Certainly, future studies assessing endocan levels in microvascular complications are needed to provide better insight into the use of endocan as a prognostic biomarker in diabetes. Poor glycemic control in diabetic patients is also related to more complications, and it seems that endocan levels decrease with improvement in glycemic control [14]. This finding suggests endocan as a potentially useful marker for monitoring glycemic control along with other traditional markers such as HgA1c.

Our study is the first systematic review and meta-analysis comparing the levels of endocan in diabetic patients with non-diabetic controls and investigating the correlation of endocan levels with complications in diabetic patients. While this systematic review can provide useful information about the role of endocan in diabetes and its complications, it has some limitations. The heterogenicity between the studies was high and was not reduced after excluding the studies with comorbidities other than diabetes. Besides, endocan levels could be elevated in many other diseases, and more studies are required to investigate its diagnostic ability in diabetes. Moreover, due to the low number of studies, we were unable to conduct a meta-analysis on the association of endocan levels with complications in diabetic patients.

## Conclusion

In general, endocan is a biomarker that is overexpressed in diabetes, regardless of the presence of other comorbidities. Additionally, our review revealed that endocan can be associated with complications of diabetes such as diabetic nephropathy and neuropathy. As endocan is a factor of endothelial dysfunction, further studies are warranted to assess its role in the pathophysiology of diabetic complications and investigate its diagnostic and prognostic role in diabetes.

## Electronic supplementary material

Below is the link to the electronic supplementary material.

**Supplementary Table 1**. Search details. **Supplementary Table 2**. Quality Assessment based on the Newcastle-Ottawa Scale (NOS). **Supplementary Table 3**. Qualities of cross-sectional studies based on Downs and Black criteria. **Supplementary Figure 1**. Funnel plot for the meta-analysis of endocan levels in diabetes. **Supplementary Figure 2**. Bubble plot for meta-regression based on mean age. **Supplementary Figure 3**. Bubble plot for meta-regression based on publication year. **Supplementary Figure 4**. Bubble plot for meta-regression based on HbA1C levels in diabetic patients. **Supplementary Figure 5**. Bubble plot for meta-regression based on the male percentage. **Supplementary Figure 6**. Bubble plot for meta-regression based on the sample size.

## Data Availability

Not applicable.
