# Supplementary material for: Endocan in prediabetes, diabetes, and diabetes-related complications: a systematic review and meta-analysis
Source: Diabetol Metab Syndr. 2023 May 16;15:102. doi: 10.1186/s13098-023-01076-z (PMC10186635; doi:10.1186/s13098-023-01076-z)
Supplement: Supplementary file 1 — Supplementary Table 1. Search details. Supplementary Table 2. Quality Assessment based on the Newcastle-Ottawa Scale (NOS). Supplementary Table 3. Qualities of cross-sectional studies based on Downs and Black criteria. Supplementary Figure 1. Funnel plot for the meta-analysis of endocan levels in diabetes. Supplementary Figure 2. Bubble plot for meta-regression based on mean age. Supplementary Figure 3. Bubble plot for meta-regression based on publication year. Supplementary Figure 4. Bubble plot for meta-regression based on HbA1C levels in diabetic patients. Supplementary Figure 5. Bubble plot for meta-regression based on the male percentage. Supplementary Figure 6. Bubble plot for meta-regression based on the sample size. [file 13098_2023_1076_MOESM1_ESM.docx]

Supplementary Materials

**Endocan in Prediabetes, Diabetes, and Diabetes-related Complications: A Systematic Review and Meta-analysis**

***Supplementary Table 1.*** *Search details*

| **Query** | | **Results**  **(13 February 2023)** |
| --- | --- | --- |
| **PubMed** | | |
| #1 | (“diabetes”[tiab] OR “diabetic”[tiab] OR “pre-diabetes”[tiab] OR “prediabetes”[tiab] OR “pre diabetes”[tiab] OR “prediabetic”[tiab] OR “pre-diabetic”[tiab] OR “pre diabetic”[tiab] OR “Diabetes Mellitus”[Mesh]) | 816,783 |
| #2 | ("Endocan" OR "ESM-1"[tiab] OR "ESM1"[tiab] OR "ESM 1"[tiab] OR "endothelial cell-specific molecule 1" OR "ESM1 protein, human" [Supplementary Concept]) | 715 |
| #3 | #1 AND #2 | 53 |
| **Scopus** | | |
| #1 | (TITLE-ABS-KEY(“diabetes”) OR TITLE-ABS-KEY(“diabetic”) OR TITLE-ABS-KEY(“pre-diabetes”) OR TITLE-ABS-KEY(“prediabetes”) OR TITLE-ABS-KEY(“pre diabetes”) OR TITLE-ABS-KEY(“prediabetic”) OR TITLE-ABS-KEY(“pre-diabetic”) OR TITLE-ABS-KEY(“pre diabetic”)) | 1,196,711 |
| #2 | (TITLE-ABS-KEY("Endocan") OR TITLE-ABS-KEY("ESM-1") OR TITLE-ABS-KEY("ESM1") OR TITLE-ABS-KEY("ESM 1") OR TITLE-ABS-KEY("endothelial cell-specific molecule 1")) | 954 |
| #3 | #1 AND #2 | 91 |
| **Web of Science** | | |
| #1 | (TS=(“diabetes”) OR TS=(“diabetic”) OR TS=(“pre-diabetes”) OR TS=(“prediabetes”) OR TS=(“pre diabetes”) OR TS=(“prediabetic”) OR TS=(“pre-diabetic”) OR TS=(“pre diabetic”)) | 828,271 |
| #2 | (TS=("Endocan") OR TS=("ESM-1") OR TS=("ESM1") OR TS=("ESM 1") OR TS=("endothelial cell-specific molecule 1")) | 913 |
| #3 | #1 AND #2 | 66 |
| **Embase** | | |
| #1 | ((“diabetes”:ti,ab,kw) OR (“diabetic”:ti,ab,kw) OR (“pre-diabetes”:ti,ab,kw) OR (“prediabetes”:ti,ab,kw) OR (“pre diabetes”:ti,ab,kw) OR (“prediabetic”:ti,ab,kw) OR (“pre-diabetic”:ti,ab,kw) OR (“pre diabetic”:ti,ab,kw)) | 1,147,417 |
| #2 | (‘Endocan’:ti,ab,kw or ‘ESM-1’:ti,ab,kw OR ‘ESM 1’:ti,ab,kw OR ‘ESM1’:ti,ab,kw OR ‘endothelial cell-specific molecule 1’:ti,ab,kw) | 1,009 |
| #3 | #1 AND #2 | 93 |
| **Total records** | | **303** |
| **Total records without duplicates** | | **172** |

***Supplementary Table 2****. Quality Assessment based on the Newcastle-Ottawa Scale (NOS)*

| **Study** | **Selection** | | | | **Comparability** | **Outcome** | | **Overall**  **Score** |
| --- | --- | --- | --- | --- | --- | --- | --- | --- |
|  | **Representation** | **Sample size** | **Non-Respondents** | **Exposure** |  | **Outcome** | **Statistical test** |  |
| Arman et al. (2015) | * | * | * | ** | - | ** | * | 8 |
| Bingol et al. (2016) | * | * | * | ** | - | ** | * | 8 |
| Chang et al. (2021) | * | * | * | ** | - | ** | * | 8 |
| Kim et al. (2020) | * | * | * | ** | - | ** | * | 8 |
| Kosir et al. (2019) | * | * | * | ** | - | ** | * | 8 |
| Moin et al. (2022) | * | * | * | ** | - | ** | * | 8 |
| Singh et al. (2022) | * | * | * | ** | - | ** | * | 8 |
| Zuwala-Jagiello et al. (2019) | * | * | * | ** | - | ** | * | 8 |

***Supplementary Table 3.*** *Qualities of cross-sectional studies based on Downs and Black criteria*

| **Study (year)** | **Reporting** | | | | | | **External Validity** | | **Internal Validity (bias)** | | **Internal Validity-Confounding (selection bias)** | | **Total Score** |
| --- | --- | --- | --- | --- | --- | --- | --- | --- | --- | --- | --- | --- | --- |
|  | **(1)** | **(2)** | **(3)** | **(6)** | **(7)** | **(10)** | **(11)** | **(12)** | **(18)** | **(20)** | **(21)** | **(22)** |  |
| Anik et al, 2020 | 1 | 1 | 1 | 1 | 1 | 1 | 1 | 1 | 1 | 1 | 0 | 0 | **10** |
| Arman et al, 2022 | 1 | 1 | 1 | 1 | 1 | 1 | 1 | 0 | 1 | 1 | 1 | 1 | **11** |
| Balamir et al, 2017 | 1 | 1 | 1 | 1 | 1 | 1 | 1 | 0 | 1 | 1 | 1 | 1 | **11** |
| Bilir et al, 2016 | 1 | 1 | 0 | 1 | 1 | 1 | 0 | 0 | 1 | 1 | 0 | 0 | **7** |
| Bozkurt et al, 2020 | 1 | 1 | 0 | 1 | 1 | 1 | 1 | 0 | 1 | 1 | 1 | 0 | **8** |
| Celik et al, 2022 | 1 | 1 | 1 | 1 | 1 | 0 | 1 | 0 | 1 | 1 | 1 | 0 | **9** |
| Chen et al, 2022 | 1 | 1 | 1 | 1 | 1 | 1 | 1 | 0 | 1 | 1 | 0 | 0 | **9** |
| Cikrikcioglu et al, 2022 | 1 | 1 | 1 | 1 | 1 | 1 | 1 | 0 | 1 | 1 | 1 | 1 | **11** |
| Dallio et al, 2017 | 1 | 1 | 1 | 1 | 1 | 1 | 1 | 0 | 1 | 1 | 0 | 0 | **9** |
| Ekiz-Bilir et al, 2019 | 1 | 1 | 1 | 1 | 1 | 1 | 1 | 0 | 1 | 1 | 1 | 0 | **10** |
| Elkamshoushi et al, 2018 | 1 | 1 | 1 | 1 | 1 | 1 | 1 | 0 | 1 | 1 | 1 | 0 | **10** |
| Klisic et al, 2020 | 1 | 1 | 1 | 1 | 1 | 1 | 1 | 0 | 1 | 1 | 1 | 1 | **11** |
| Klisic et al. (2), 2020 | 1 | 1 | 1 | 1 | 1 | 1 | 1 | 0 | 1 | 1 | 0 | 0 | **9** |
| Kose et al, 2015 | 1 | 1 | 1 | 1 | 1 | 0 | 1 | 0 | 1 | 1 | 1 | 0 | **9** |
| Lv et al, 2017 | 1 | 1 | 1 | 1 | 1 | 1 | 1 | 0 | 1 | 1 | 1 | 1 | **11** |
| Qiu et al, 2016 | 1 | 1 | 1 | 1 | 1 | 0 | 1 | 0 | 1 | 1 | 1 | 1 | **10** |

Abbreviation: (0) No / Unable to determine; (1) Yes

(1) Is the hypothesis/aim/objective of the study clearly described? (2) Are the main outcomes to be measured clearly described in the Introduction or Methods section? (3) Are the characteristics of the patients included in the study clearly described? (6) Are the main findings of the study clearly described? (7) Does the study provide estimates of the random variability in the data for the main outcomes? (10) Have actual probability values been reported (e.g., 0.035 rather than < 0.05 for the main outcomes except Where the probability value is less than 0.001? (11) Were the subjects asked to participate in the study representative of the entire population from which they were recruited? (12) ﻿Were those subjects who were prepared to participate representative of the entire population from which they were recruited? (18) Were the statistical tests used to assess the main outcomes appropriate? (20) Were the main outcome measures used accurate (valid and reliable)? (21) Were the patients in different intervention groups (trials and cohort studies) or were the cases and controls (case‐control studies) recruited from the same population? (22) Were study subjects in different intervention groups (trials and cohort studies) or were the cases and controls (case‐control studies) recruited over the same period of time?

^*^High methodological quality (score >8), medium quality (score 4-8), low quality (score <4).

**
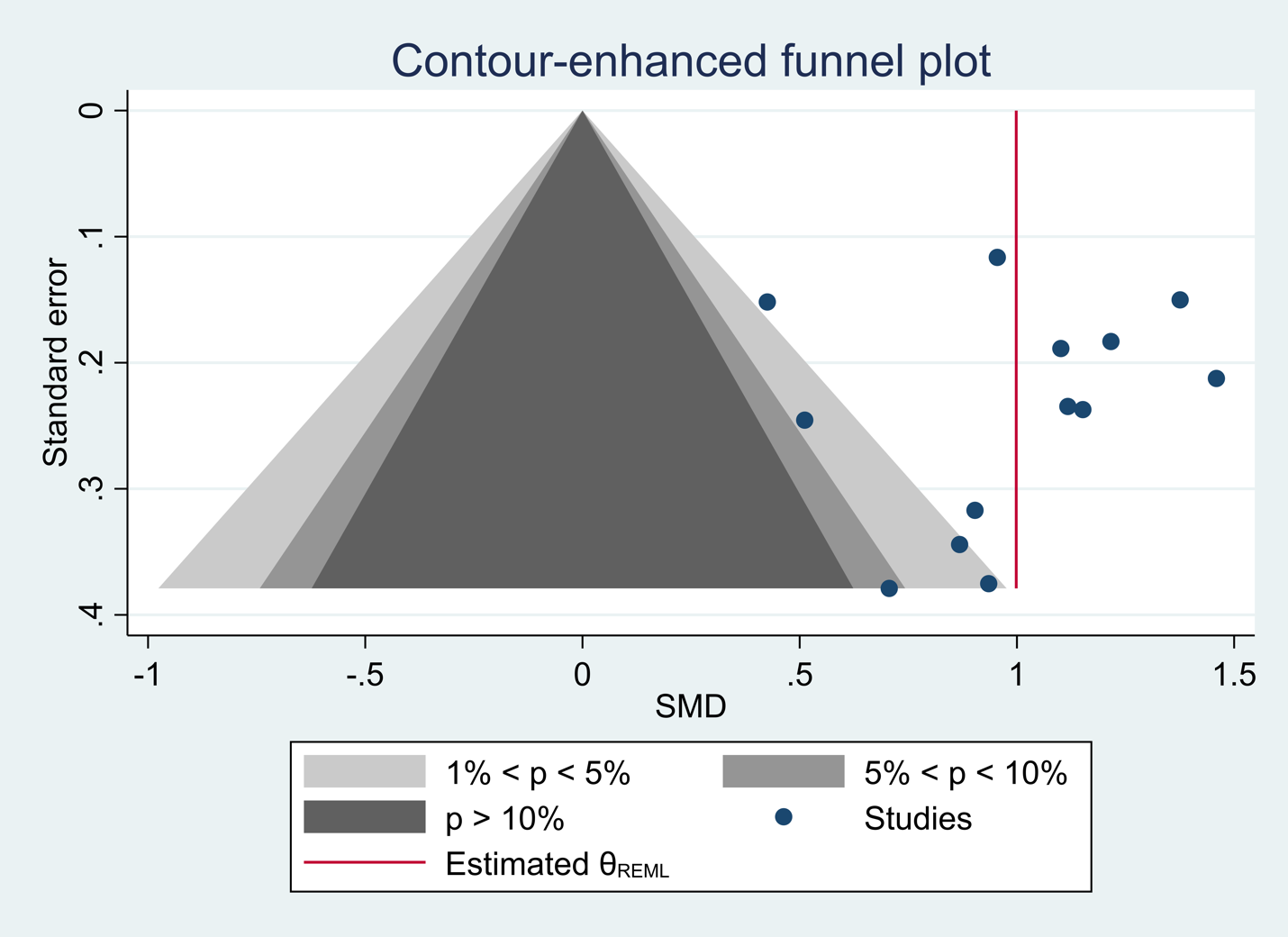
**

***Supplementary Figure 1.*** *Funnel plot for the meta-analysis of endocan levels in diabetes*

*
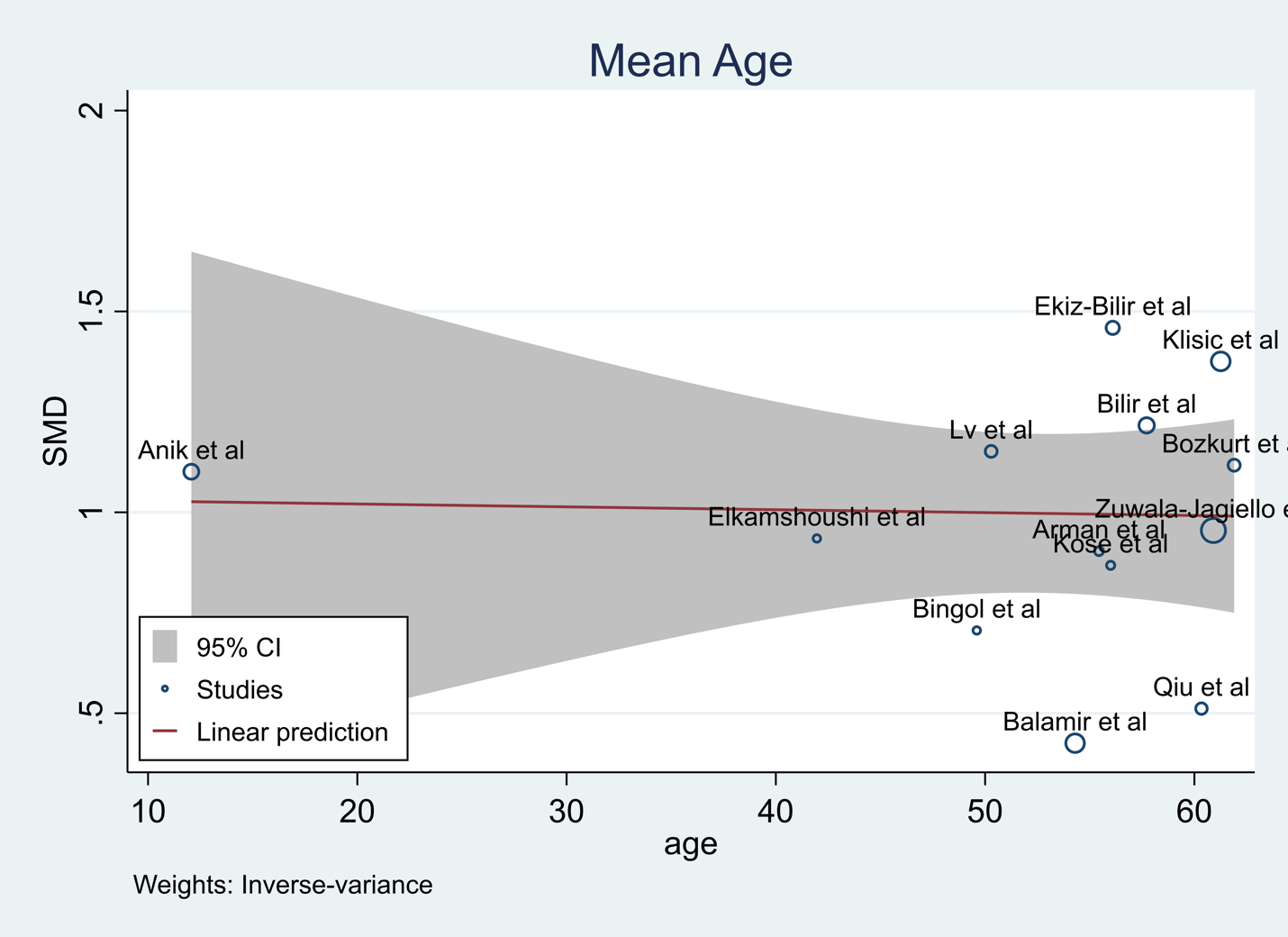
****Supplementary Figure 2.*** *Bubble plot for meta-regression based on mean age*


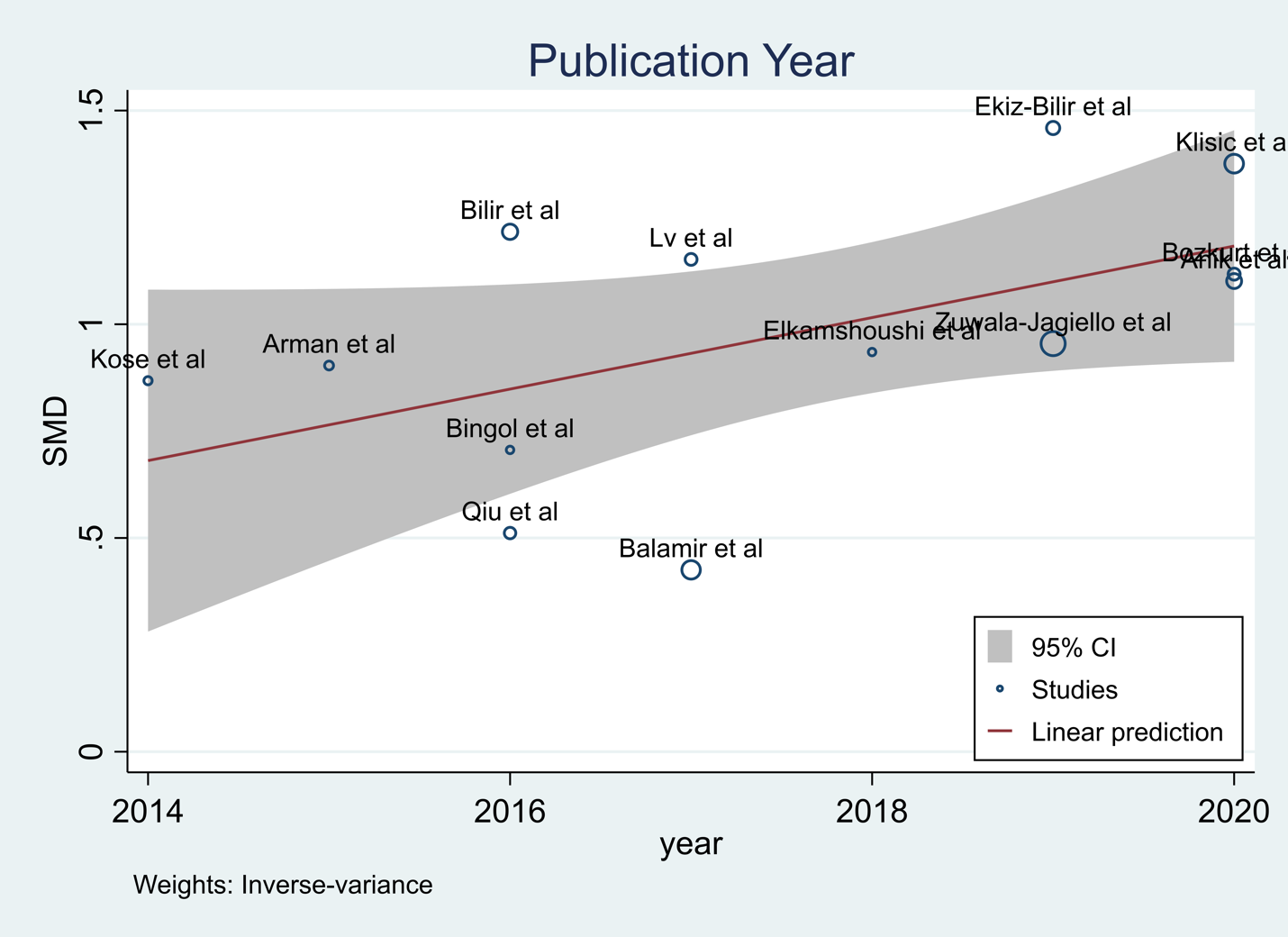


***Supplementary Figure 3.*** *Bubble plot for meta-regression based on publication year*


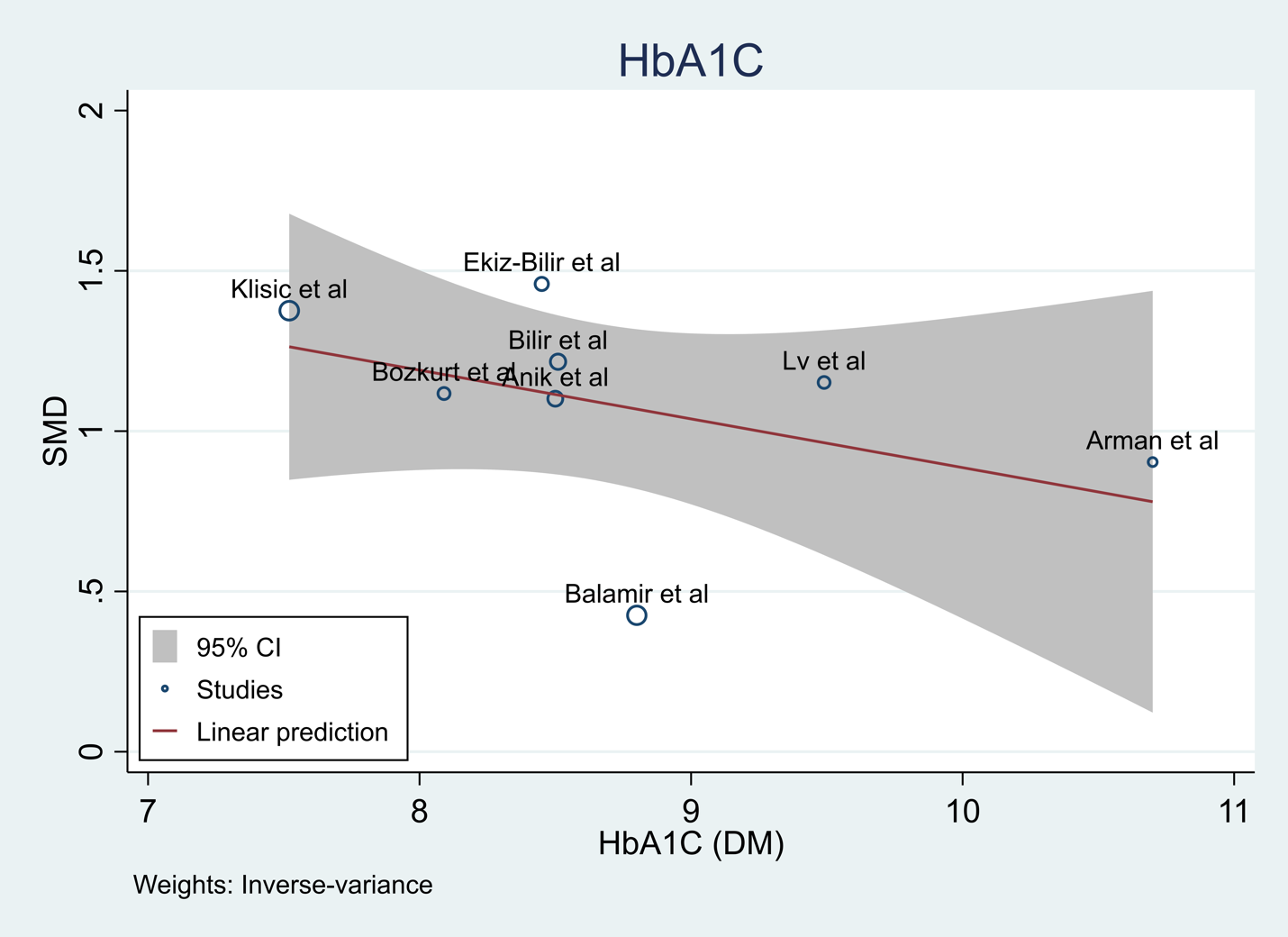


***Supplementary Figure 4.*** *Bubble plot for meta-regression based on HbA1C levels in diabetic patients*


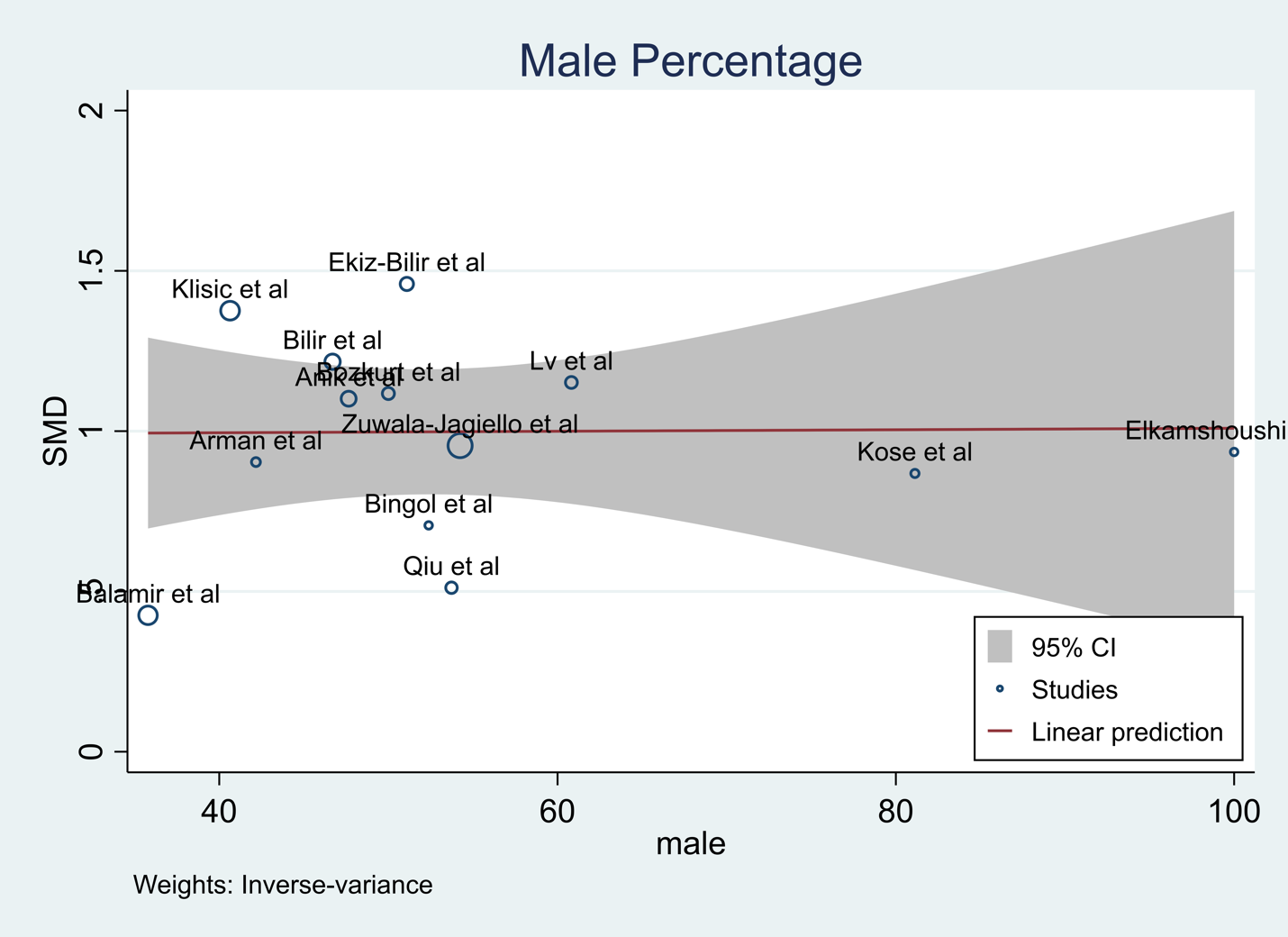


***Supplementary Figure 5.*** *Bubble plot for meta-regression based on the male percentage*


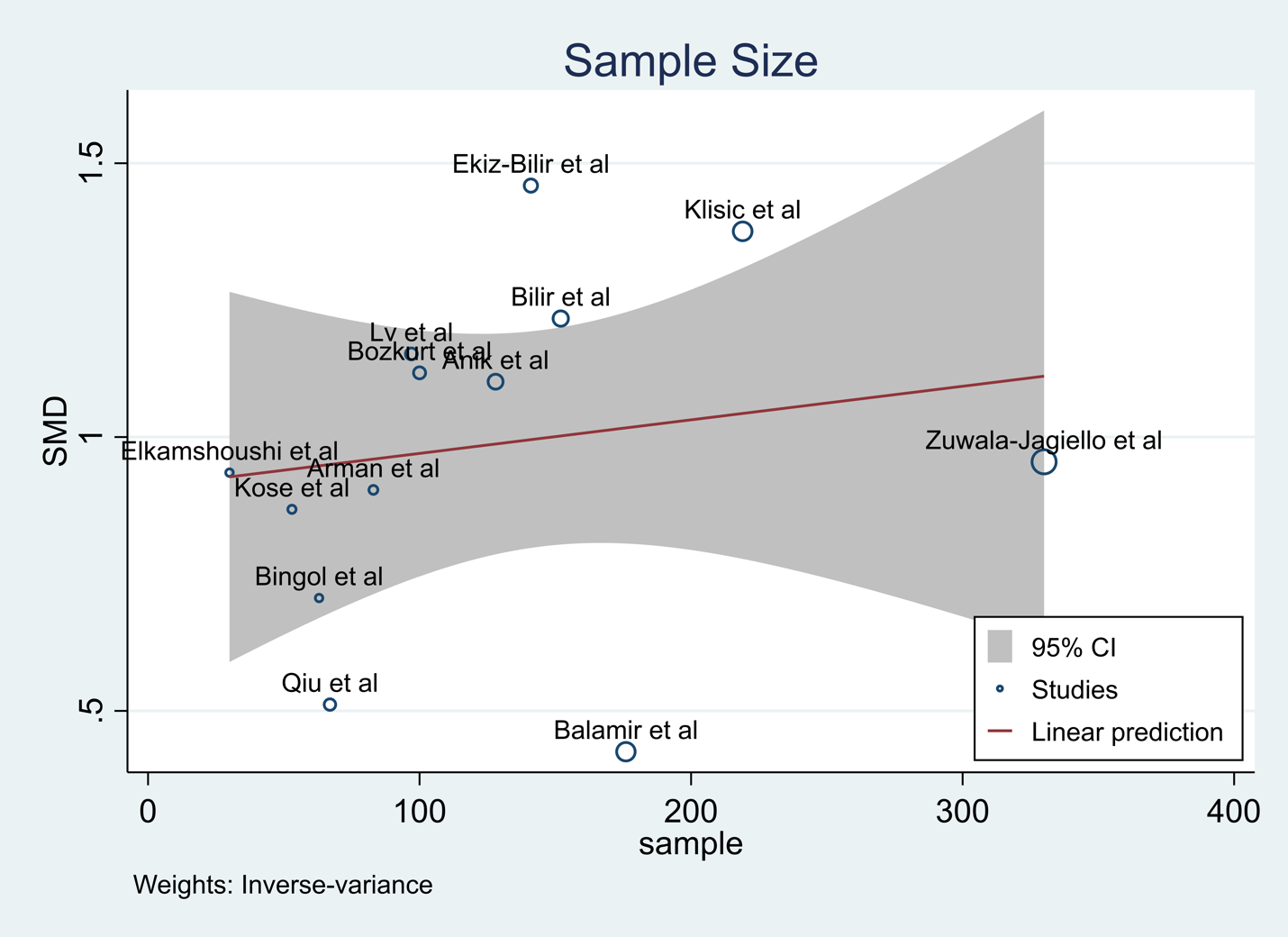


***Supplementary Figure 6.*** *Bubble plot for meta-regression based on the sample size*
